# Progress in Medicine

**Published:** 1894-11-24

**Authors:** 


### Progress in Medicine.

### INFECTIVE DISEASES.

(*Continued from page* 118.)

**Small Pox.**—Opinions vary much as to whether the microbial cause of the disease will prove to be of vegetable origin or a protozoon. A parasitic round body four times the size of a staphylococcus has been found by many observers of late in the pustules of vaccinia and variola, which is transmitted by inoculation. Ruffer and Plimmer describe the epithelial cells around it as undergoing degenerations similar to those seen in cancer, but nothing is known of its importance in the disease.[1] Stephen C. Martin isolated and obtained cultures of a bacterium which gave cow pox to calves, and in the fourteenth generation of the culture he obtained from it a typical vesicle on a child.[2] Of course, confirmation of this will be eagerly looked for.

Monkton Copeman, at the Bristol meeting,[3] considered Ruffer's cocci as probably only the products of irritation, while he and Klein find a bacillus in the lymph at an early stage. This can also be demonstrated in the tissues, but cannot as yet be cultivated in artificial media like the numerous saprophytes found in the lymph. This bacillus is about twice as long as broad, and the two ends stain deeply. He claimed that all observers except Chauveau agreed that vaccinia could be produced by inoculation of the cow with variola. Calves are the most susceptible, and Chauveau possibly failed by taking adult animals for his experiments. Klein has succeeded in inoculating animals with variola, and found them immune to the ordinary lymph. This also is confirmed by Monkton.

Voigt of Hamburg in a most important *résumé* of the history of the production of vaccinia by inoculation of cows with variola said that he had twice succeeded in obtaining good vesicles. The lymph does not become fit for use on human beings until it has been passed through several animals, and enormous numbers of people in England and the Continent have been vaccinated with matter originally derived in this way from small-pox. He showed how impossible it is that this vaccine had resulted from accidental infection with old vaccine at the time when the animals were inoculated with small-pox, seeing also that the new stock is stronger than any which results from common vaccine. The unity of the virus of variola and vaccinia was strongly insisted upon by him and by Drs. Hime and Cornish. Hime showed that vaccinia is transformed variola, and never reverts, losing the double fever, having a shortened life, and ceasing to be infectious through the air or to be productive of vesicles away from the inoculated spot. The single instance of failure to effect this transformation was that of Chauveau; though the passage of variola through a single animal may not suffice to do this, yet it is abundantly proved that absolutely normal vaccine has been derived over and over again from stocks descended from variola. Thus half-a-million of people in India were vaccinated from Dr. King's transformed variola.

However, the advocates of the duallist theory find support in the failures of the late Juhel Renoy to cultivate variola in cattle in some recent elaborate experiments which, he considered, confirmed the views of Chauveau. Hervieux points out[4] the variability of variola and the stability of vaccine, which never reverts to the variolous type, and the uniform benignity of vaccine while retaining its protective power, which contrasts strongly with the action of an attenuated virus. Chantemesse, in reply, agreed that the two poisons, as seen in human beings, are perfectly distinct, but the question of a common origin depends on experiments, and the evidence is strong that some investigators have succeeded in variolous inoculations, and have obtained stocks of ordinary vaccine from this source. The few experiments of Renoy and Chauveau cannot entirely outweigh all this evidence.

The results of vaccination by grattage are shown to have been unreliable in many cases in Mexico by Ortega[5], while the large abraded surface leaves severe scars, and much septic invasion takes place.

Hochsinger recently advocated the old German view that varicella and variola were identical, but evidence to disprove it comes from all quarters. Vetchtomoff[6] mentions two children who had smallpox while their two brothers escaped by vaccination, and all four took varicella, though, on the other hand, André Martin[7] mentions two girls who came to a house where varicella existed. One of them developed varicella and the other variola; a month later those who nursed them developed varicella. Talamon suggests that by contact with vaccinated persons one who has partly lost his vaccine immunity may develop varicella, but Œttinger[8] shows that numbers of convalescents from varicella have been successfully vaccinated, and relates a case of a child recovering from varicella who took small-pox, and being vaccinated too late ran through vaccinia also simultaneously.

In another paper he discusses the value of the bed-chamber method of treating small-pox.[9] He treated eight very severe cases by this plan from an early stage. The mortality was not reduced, but the vesico-pustules dried up, pitting was prevented, and the secondary fever of suppuration was minimised. Feilberg's pamphlet explaining the method is summarised by Moir,[10] who does not, however, believe in its utility. Gama at Rome insisted on the value of sulphide of calcium,[11] which with carbolate of camphor ointment is also praised by T. A. Elder.[12] The pathology and bacteriology of the broncho-pneumonia of small-pox cases is detailed by Auché,[13] who regards it as due to a secondary infection, by pneumococci, or strepto and staphylo-cocci. In another paper,[14] he discusses the influence of small-pox in the parents on the immunity of their children. Recent small-pox in the mother prior to or during pregnancy sometimes protects, but often does not. There is no invariable rule. Dr. Walter Dowson brings forward many facts to show that the lung affection is primary, and that small-pox is, in fact, only an infective pneumonia. The resemblance of the initial stages to those of pneumonia,[15] the laryngeal troubles, the skin eruption, and the infection through expired air are brought forward among other points, as tending to show that the primary lesion is in the lungs.

The aerial diffusion of small-pox has continued to excite much attention. In Vienna and Buda Pesth, however, the new hospitals are built close to large general infirmaries,[16] so that English views do not appear to be accepted there. Dr. Priestly, who was formerly opposed to the theory of aërial diffusion, acknowledges his conversion from the facts he observed during the late Leicester epidemic.[17] The infection may be through sewer air, winds, and also through rats and flies. Dr. Alfred Hill said that the Birmingham experience was against aërial convection, and there the workhouse, asylum, and gaol were close to the small-pox hospital. Similar evidence from Portsmouth, Coventry, and Birkenhead was given in the discussion, but from Warrington,[18] Crediton, and Lancashire instances of infection of surrounding districts were given, though whether this took place by aerial convection or by personal contagion was not absolutely certain. The absence of diffusion from the Gore Farm Hospital cannot be explained by the patients being all in the convalescent stage,[19] for owing to the need of room many were sent there in comparatively early stages of the disease; but an outbreak at Solihull[20] was, in the opinion of the health officers, caused by infected steam from the laundry of the smallpox hospital, thirty feet away. The result of the Bradford epidemic was detailed at Bristol by Dr. Evans, who concluded that the poison was carried aërially from the wards of the hospital, as the evidence against personal contagion was very strong.[21] The position of outbreaks, moreover, varied remarkably with the prevalent winds.

The lesson of the Leicester epidemic has been an important one. Infant vaccination has in other towns caused most attacks to occur in adults, but Leicester stands alone in the proportion of children who suffered.[22] One hundred and seven cases were found in children out of 347; only two abortive ones being in vaccinated children. The mortality in certainly vaccinated adults was only ·5 per cent., but in the unvaccinated 8·3 cent. Again, the official report mentions that among 1,261 "quarantined" persons who were exposed to infection only 6·2 per cent. vaccinated took the disease, but 21·5 per cent. of the unprotected. Of the nurses and attendants no efficiently protected person took the disease, while of six vaccinated only in infancy five suffered. Similarly at Sheffield, of 81 revaccinated nurses not one was affected, but of 62 once vaccinated six took the disease, and in London of 645 revaccinated not one suffered, while every one of the ten others was attacked.[23] Dr. Priestly further holds that quarantining can be carried out most efficiently and cheaply at the patients' own houses, indeed accommodation otherwise soon failed in even this moderate outbreak. In the absence of vaccination an enormous amount of compulsory notification and quarantining would be necessary. Layet, of Bordeaux, in 2,000 cases found that the mortality was five times less in vaccinated children than in the unvaccinated, and in revaccinated persons under 10 no death occurred.[24]

As to the effect of typhoid and malarial fever in destroying immunity after vaccination or small-pox, Dr. Pinder finds that this is usually the case,[25] and such persons can generally be successfully revaccinated. He had 103 cases in which a second vaccination took.

In view of the vast amount of inefficient vaccination and the evasion of it altogether, Dr. Yarrow proposes[26] to allow every registered practitioner to claim a fee of 2s. 6d. from the rates for each thorough vaccination, subject to due inspection. It is needless to discuss the difficulties of this course, but a vast amount of danger is being caused by the notoriously inefficient vaccination in one small spot, which is so common in poorer districts.

Dr. Boobyer, in an interesting paper on the history of small-pox, draws[27] attention to the frequency of prodromal rashes, especially in modified cases, and to the importance of a hitherto undescribed diagnostic mark of small-pox in the presence of polished, circular, often rayed scabs, found chiefly on the upper extremities even in modified attacks. These could be seen forming at a very early stage, by the aid of a glass, in the abortive vesicles and pocks.

The anti-vaccinationists of Leicester claim that among the sources of infection were very few unvaccinated persons, forgetting that the large majority of the people are still vaccinated; even 25 per cent. of the quarantined children were so. Besides, the disease in unvaccinated persons is at once recognised,[28] while it is the modified undistinguishable cases which are passed over, and which spread the disease.

The low death-rate generally of the recent epidemic in Leicester is hardly an argument against vaccination, but rather shows the skill in treatment. In fact, the death-rate from small-pox in 1871-2 for all England was 900 per million, and in 1892-3 only 32 per million. In London the fall was 50 to one, while in Leicester it was only 40 to one. According to the *British Medical Journal*,[29] Mr. Biggs further spoke of the protected population of Leicester as only 10,000 to 174,000 unprotected, because he finds a statement that infantile vaccination loses much of its force after ten years. He then gives to the 10,000 all the 178 cases of small-pox which occurred in vaccinated persons, though 176 were over that age. Moreover, the use of the hospital for small-pox resulted in leaving at home the scarlet fever patients, which flourished to the number of 4,000 cases in the year. In Leicester only one out of 178 cases of small-pox in the vaccinated died, against 19 out of 153 unvaccinated ones. The evidence of Professor Crookshank before the commission contains the statement that the effect which cow-pox has upon small-pox, "if such exist at all, is a very transient antagonism, and not a specific protection," and "even variola vaccine produces a very transient effect." He recommends a compulsory system of notification and isolation.[30] Much surprise has been felt at this as coming from a man of his scientific training; but in spite of his strong belief that vaccinia has nothing to do with variola, he acknowledges that vaccination as now practised does protect many persons for a time.[31] Even the *Homœopathic Review* has a strong article on the Leicester epidemic, as showing "the well substantiated fact" of the protection afforded by vaccination, and its immense value to all exposed to the infection of small-pox.[32] From America we have a critical record of 5,000 cases of small-pox treated by Dr. Welch in the Philadelphia Hospital.[33] He finds that there is no difference in the death-rate between the sexes, nor in that of unvaccinated negroes and whites so far as his observations go. The unvaccinated yielded a mortality of 58·3 per cent. and the vaccinated 16·2, those showing fair cicatrices having only 10·6 per cent. and those with poor ones 27·1 per cent. All the rates are high from causes he mentions. The mortality among the unvaccinated again was highest among the Irish patients, and about equal in Germans and Americans. The German vaccinations appeared more uniformly protective, but Dr. Welch could not find any relation between the number of good cicatrices and the degree of protection, contrary to the statistics of Marson. He confirms the ordinary views of the value of infantile and subsequent revaccination. Seventy-eight cases occurred after alleged revaccination, most of them very mild, but no instance occurred among previously revaccinated nurses in 23 years. Forty-five alleged cases of a second attack of small-pox were noted, and of these most were of a mild type. The report is altogether of great value and completeness. A summary of the 1893 epidemic in eleven great English towns shows that of 2,177 vaccinated cases only 4·1 per cent. died, while of 374 unvaccinated, 33·9 per cent. perished, in other words, that there were 122 people killed by neglect of vaccination. In children under 10 the mortality of the unvaccinated was 22 times greater than that of the vaccinated.

[1] B. M. J., June 30. [2] Med. Rec., June 2. [3] B. M. J., Sept. 22. [4] Med. Week, Feb. 23 [5] Am. J. Med. S., Sept. [6] B. J. Dermatol., March. [7] Practit., June. [8] Med. Week, Feb. 10. [9] Med. Week, June 1. [10] Lancet, Sept. 29. [11] Med. Rec., April 14. [12] N. Y. Med. J., Ap. 28. [13] Int. Med. Mag., June. [14] Int. Med. Mag., Sept. [15] Public Health, June. [16] B. M. J., Sept. 29. [17] Lancet, Aug. 4. [18] Practit., June. [19] Lancet, July 14. [20] Lanc., Ap. 14. [21] B. M. J., Aug. 18. [22] Practit., June. [23] B. M. J., May 12. [24] Lanc., June 3. [25] Med. Rec., Feb. 10. [26] Lancet, May 19. [27] Lancet, May 26. [28] Lancet, June 30. [29] B. M. J., July 31. [30] Lancet, June 23. [31] B. M. J., July 14. [32] Homœopath. Review, Aug. [33] N. Y. M. J., March 17. [34] B. M. J., Ap. 21.

